# Preventive dental care reduces risk of cardiovascular disease and pneumonia in hemodialysis population: a nationwide claims database analysis

**DOI:** 10.1038/s41598-024-62735-3

**Published:** 2024-05-29

**Authors:** Risako Mikami, Koji Mizutani, Miho Ishimaru, Tomohito Gohda, Takanori Iwata, Jun Aida

**Affiliations:** 1 Department of Periodontology, Graduate School of Medical and Dental Sciences, Tokyo Medical and Dental University (TMDU), Tokyo, Japan; 2 Institute of Education, Tokyo Medical and Dental University (TMDU), 1-5-45 Yushima, Bunkyo-ku, Tokyo, 113-8549 Japan; 3 Department of Nephrology, Juntendo University Faculty of Medicine, Tokyo, Japan; 4 Department of Oral Health Promotion, Graduate School of Medical and Dental Sciences, Tokyo Medical and Dental University (TMDU), Tokyo, Japan

**Keywords:** Kidney failure, Renal dialysis, Dental care, Big data, Communicable diseases, Cardiovascular diseases, Dentistry, Renal replacement therapy

## Abstract

This study aims to investigate the impact of dental care utilization status on the occurrence of fatal complications such as cerebral/cardiovascular disease (CVD) and infectious diseases in patients with end-stage renal disease (ESRD) undergoing hemodialysis. This retrospective cohort study was performed using the Japanese claims database and included patients who first underwent hemodialysis between April 2014 and September 2020. The exposure variable of interest was the pattern of dental utilization, which was categorized into three groups, “dental treatment group”, “preventive dental care group”, and “no-dental visit group”. The primary outcomes were the time interval until a composite end point of first major cardiovascular event (acute myocardial infarction, heart failure, or cerebral infarction) of infectious disease (pneumonia and sepsis). The secondary outcomes were the time interval until the incidence of each component of primary outcomes. Survival analyses, including log-rank tests and Cox proportional hazards regression analyses, were performed. Among the 10,873 patients who underwent the first dialysis treatment, 6152 were assigned to the no-dental visit group, 2221 to the dental treatment group, and 2500 to the preventive dental care group. The preventive dental care group had significantly lower hazard ratios (HRs) of the incidence of CVD (adjusted hazard ratio [aHR]: 0.86, 95% confidence interval [CI]: 0.77–0.96) and infectious diseases (aHR: 0.86, 95% CI: 0.76–0.97). As for pneumonia, preventive dental care and dental treatment groups had significantly lower HRs (aHR: 0.74 and 0.80, 95% CI: 0.61–0.88, 0.66–0.96) than the no-dental visit group. This study demonstrated that dental visits for preventive dental care were associated with a significant risk reduction in CVD and infectious complications in patients with ESRD undergoing hemodialysis.

## Introduction

Chronic kidney disease (CKD) is a tremendous burden on global health, both as a direct cause of morbidity and mortality worldwide and as an important risk factor for the incidence of cerebral/cardiovascular disease (CVD) and infectious diseases^1^. Hemodialysis is a renal replacement therapy for patients with end-stage renal disease (ESRD). Patients undergoing hemodialysis have a significantly reduced quality of life (QoL), high medical costs, and a high crude mortality rate of > 20% per year^2^. CVD and infectious diseases are the major causes of death among patients undergoing hemodialysis, accounting for 50%^3^ and 14% of deaths, respectively^4^.

Oral health status influences mortality in patients undergoing hemodialysis. Several studies have demonstrated the effect of periodontitis on mortality in patients undergoing hemodialysis^5–8^. Furthermore, dental caries^9^, decayed-missing-filled teeth index^10^, and poor oral hygiene^9,10^ were associated with mortality in patients undergoing hemodialysis^9^. Previous reports have also revealed the dental-related diseases with major causes of death under hemodialysis maintenance; for example, between dental caries and aortic calcification^11^, as well as between oral candidiasis and infectious diseases^12^. Recent studies have emphasized the effectiveness of periodontal therapy in suppressing fatal events such as CVD^3^, infectious disease^13^, and mortality^3^ in patients undergoing hemodialysis. However, no studies have examined the impact of dental interventions other than periodontal therapy in patients undergoing hemodialysis. Therefore, we hypothesized that general dental care and treatments, such as dental caries treatment, prosthodontic treatment, tooth extraction, and periodontal therapy, and the preventive dental care may reduce the incidence of fatal complications such as CVD and infectious disease in patients undergoing hemodialysis.

This study aimed to investigate the impact of dental care utilization status on the occurrence of fatal complications such as CVD and infectious diseases in patients undergoing hemodialysis using the Japanese claims database.

## Methods

### Data source

This retrospective cohort study used data from the DeSC database, which contains claims data provided by DeSC Healthcare Inc., Tokyo, Japan (https://desc-hc.co.jp/company)^14^. This database includes individuals insured with the following three types: (1) society-managed, employment-based health insurance associations (provided for employees of Japanese companies and their families), (2) national health insurance (provided for individuals aged < 75 years who are not covered by other public health insurance), and (3) the latter-stage elderly healthcare system (provided for individuals aged 75 years or more). DeSC Healthcare Inc. possesses epidemiological claims data for three million insurance subscribers. This data are available for academic research with anonymized information on medical, dental, and drug prescription claims and health check-ups^14^. A previous study reported that the distribution of age and sex in the database was comparable to that of the population estimates. Furthermore, the same study reported that the prevalence of certain non-communicable diseases in the database was comparable to that in a previously reported national survey^14^. In this claims data, all medical diagnoses were made according to the International Classification of Diseases 10th Revision (ICD-10) codes and recorded in Japanese free texts.

### Ethics statement

This study was approved and informed consent was waived by the Dental Research Ethics Committee of the Tokyo Medical and Dental University (approval number: D2022-017). This study was conducted according to the Helsinki Declaration of 1975, as revised in 2013.

### Sampled patients

**Figure 1:**
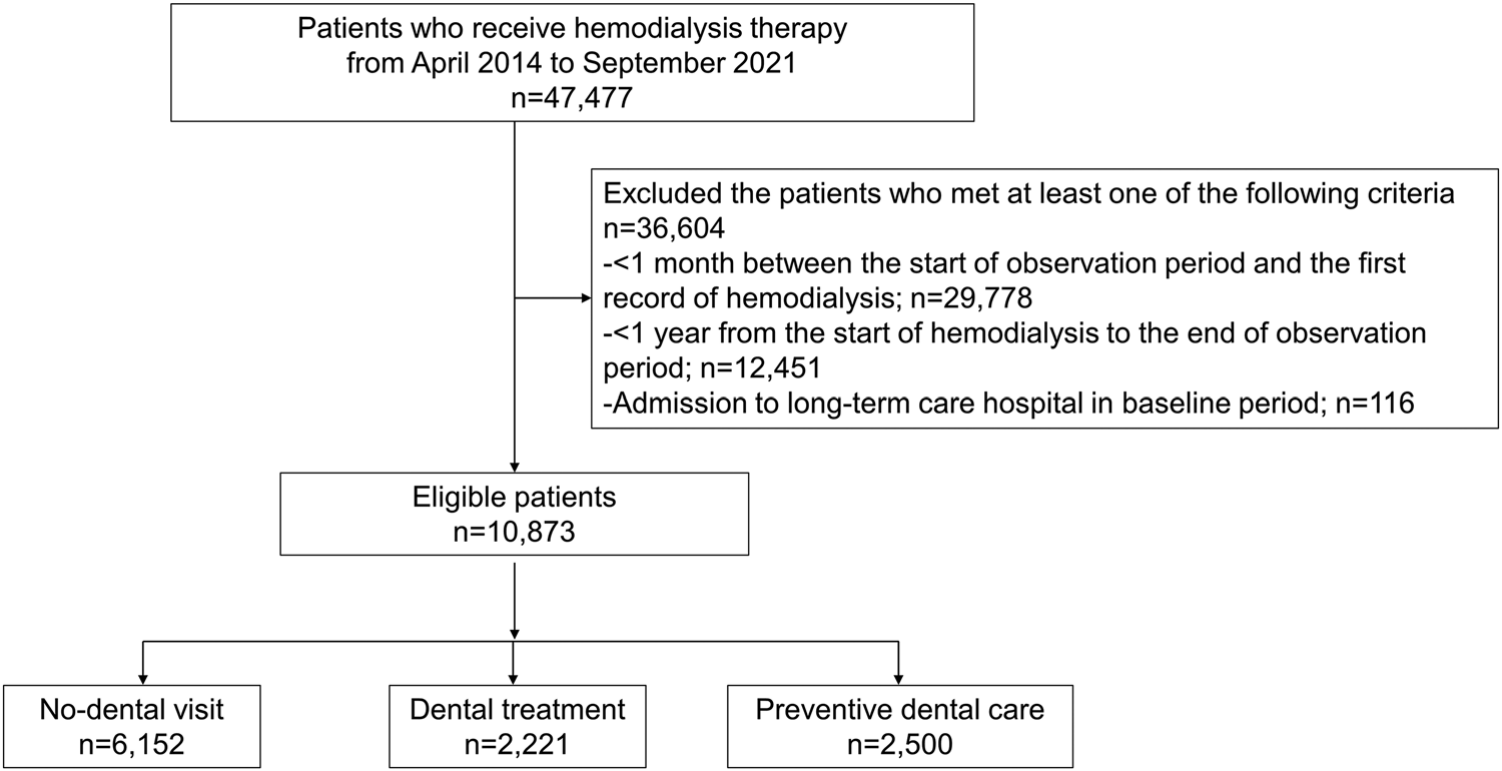
Flow diagram of patient selection.

A flowchart of the patient selection for this study is presented in Fig. 1. We identified patients who underwent hemodialysis between April 2014 and September 2020. Among them, we extracted patients who had at least 1 month between the start of the observation period and the first hemodialysis record to identify newly introduced hemodialysis patients. Patients on maintenance hemodialysis generally undergo hemodialysis three times a week. Patients who survived for at least 1 year during the follow-up period were selected to identify hemodialysis patients in the chronic maintenance period. The baseline period was defined as 1 year after the first hemodialysis. Patients admitted to hospitals requiring long-term care during the baseline period were excluded. Individuals diagnosed with each outcome during the baseline period were also excluded.

### Exposure

The exposure variable of interest in this study was the pattern of dental care utilization, which was categorized into three groups: “dental treatment group”, “preventive dental care group”, and “no-dental visit group”. In clinical practice, people who visit dentists only when they experience symptoms may discontinue their dental visit after treatment for chief complaints, even if further treatment is recommended. In contrast, some patients regularly visit dentists and continue to take preventive dental care, oral hygiene instructions, prophylaxis, and prosthesis maintenance after the symptomatic therapy. Among these people accessing dental clinics, people exist with different attributes: treatment and preventive dental care groups.

We defined patients who did not have a dental visit during the baseline period as the “no-dental visit group.” Patients who had received dental treatments, such as cavity fillings, pulpectomies, periodontal surgeries, tooth extractions, and placement of prostheses, were assigned to the “dental treatment group.” Patients who had a dental visit during the baseline period but did not receive the treatment mentioned above were assigned to the “preventive dental care group” (see Supplementary Table 1 for more details). Regarding the additional analyses conducted to examine the effect of different dental procedures, the dental treatment group was further divided into the “minor dental treatment group,” which included the patients who had undergone cavity filling and placement of prostheses, and “major dental treatment group,” which included patients who had undergone pulpectomies, periodontal surgeries, and tooth extractions.

### Outcome measurement

The primary outcome of this study was the time interval until the incidence of CVD and infectious diseases. CVD included diagnoses of acute myocardial infarction (ICD-10 code: I21), heart failure (ICD-10 code: I50), and cerebral infarction (ICD-10 code: I63) during the observation period. Infectious diseases included sepsis (ICD-10 code: A40 and 41), and pneumonia (ICD-10 code: J15 and 69). The secondary outcome was the time interval until the incidence of acute myocardial infarction, heart failure, cerebral infarction, sepsis, or pneumonia. For survival time analysis, survival time was defined as the time from the start of observation to the earliest of (1) event occurrence, (2) death, (3) censoring, and (4) the end of the observation period. Regarding the sensitivity analyses, survival time analyses for the outcome were performed, with an event defined a disease recorded more than twice during the observation period. Survival time was defined as the period from the start of observation to the time when a disease is recorded for the first time in the observation period.

### Covariates

Health and socio-demographic variables were used as covariates. Diagnoses of the following comorbidities based on the Charlson comorbidity index between baseline periods were identified: diabetes, hypertension, dementia, chronic pulmonary disease, rheumatic disease, liver disease, hemiplegia, paraplegia, malignancy, metastatic solid tumor, and human immunodeficiency virus/acquired immunodeficiency syndrome (HIV/AIDS) (see Supplementary Table 2 for more details). For the history of hospitalization in the baseline period, the patient was hospitalized for any reason during the baseline period was extracted. As a proxy for socioeconomic status, the type of health insurance was categorized as (1) society-managed employment-based health insurance associations, (2) national health insurance, and (3) the latter-stage elderly healthcare system. Age and sex were also considered.

### Statistical analysis

Survival time analyses were conducted to examine the effects of dental utilization patterns on the incidence of each outcome. The cumulative incidence of each outcome was illustrated using Kaplan–Meier curves and compared between groups using log-rank tests. Hazard ratios (HRs) and 95% confidence intervals (CIs) of the selected outcomes were estimated using Cox proportional hazard regression models. In the multivariable models, the following covariates were included: age, sex, diabetes, hypertension, congestive heart failure, dementia, chronic pulmonary disease, rheumatic disease, liver disease, hemiplegia, paraplegia, malignancy, metastatic solid tumor, HIV/AIDS, history of hospitalization in the baseline period, and type of health insurance. Schoenfeld residual plots were plotted to confirm that the proportional hazards assumption of the Cox proportional regression models was not violated for each outcome^15^ (Supplementary Fig. 1). All statistical analyses were performed using Stata software (version 17.0; StataCorp). All tests were two-tailed, and statistical significance was set at p < 0.05.

## Results

We included 47,477 patients who underwent hemodialysis during the study period. Among them, 29,778, 12,451, and 116 participants were excluded due to the duration from the start of the observation period to the first record of hemodialysis of less than 1 month, the duration from the start of hemodialysis to the end of observation period of less than 1 year, and admission to a long-term care hospital during the baseline period, respectively. We identified 10,873 as eligible patients. Of the eligible patients, 6152 (56.6%) were assigned to the no-dental visit group, 2221 (20.4%) to the dental treatment group, and 2500 (23.0%) to the preventive dental care group (Fig. 1).

The baseline characteristics of the patients are shown in Table 1. The mean age was 74.0 years (standard deviation [SD]: 12.2), with the majority being older than 65 (80.6%). Males accounted for 7190 (66.1%) of the total. Among the patients in this study, many had multiple comorbidities, and more than half were hospitalized in the baseline period.

**Table 1:** Characteristics of participants (N = 10,873).

Characteristics	All (N = 10,873)	No-dental visit (N = 6152)	Dental treatment (N = 2221)	Preventive dental care (N = 2500)
	Mean (SD), or N (%)	Mean (SD), or N (%)	Mean (SD), or N (%)	Mean (SD), or N (%)
Age (years)	74.0 (12.2)	73.8 (12.5)	73.8 (11.7)	74.7 (11.8)
≤ 65	2112 (19.4%)	1264 (20.6%)	413 (18.6%)	435 (17.4%)
65–80	4838 (44.5%)	2661 (43.3%)	1058 (47.6%)	1119 (44.8%)
≥ 80	3923 (36.1%)	2227 (36.2%)	750 (33.8%)	946 (37.8%)
Sex				
Male	7190 (66.1%)	3992 (64.9%)	1519 (68.4%)	1679 (67.2%)
Female	3683 (33.9%)	2160 (35.1%)	702 (31.6%)	821 (32.8)
Type of health insurance				
Society-managed, employment-based health insurance	347 (3.2%)	197 (3.2%)	57 (2.6%)	93 (3.7%)
National health insurance	2656 (24.4%)	2656 (24.4%)	523 (23.6%)	530 (21.2%)
The latter-stage elderly healthcare insurance	7879 (72.4%)	4352 (70.7%)	1641 (73.9%)	1877 (75.1%)
Disease history				
Diabetes	9410 (86.5%)	5355 (87.0%)	1897 (85.4%)	2158 (86.3%)
Hypertension	10,653 (98.0%)	6036 (98.1%)	2177 (98.0%)	2440 (97.6%)
Dementia	1882 (17.3%)	1091 (17.7%)	314 (14.1%)	477 (19.1%)
Chronic pulmonary disease	4099 (37.7%)	2205 (35.8%)	874 (39.4%)	1020 (40.8%)
Rheumatic disease	2645 (24.3%)	1473 (23.9%)	519 (23.4%)	653 (26.1%)
Mild liver disease	6695 (61.6%)	3761 (61.1%)	1363 (61.4%)	1571 (62.8%)
Moderate or severe liver disease	316 (2.9%)	174 (2.8%)	66 (3.0%)	76 (3.0%)
Hemiplegia or paraplegia	427 (3.9%)	253 (4.1%)	76 (3.4%)	98 (3.9%)
Malignancy	8609 (79.2%)	4768 (77.5%)	1779 (80.1%)	2062 (82.5%)
Metastatic solid tumor	1045 (9.6%)	536 (8.7%)	221 (10.0%)	288 (11.5%)
HIV/AIDS	534 (4.9%)	285 (4.6%)	96 (4.3%)	153 (6.1%)
Hospitalization in baseline period	6169 (57.0%)	3399 (55.3%)	1279 (58.6%)	1518 (60.7%)

**Figure 2:**
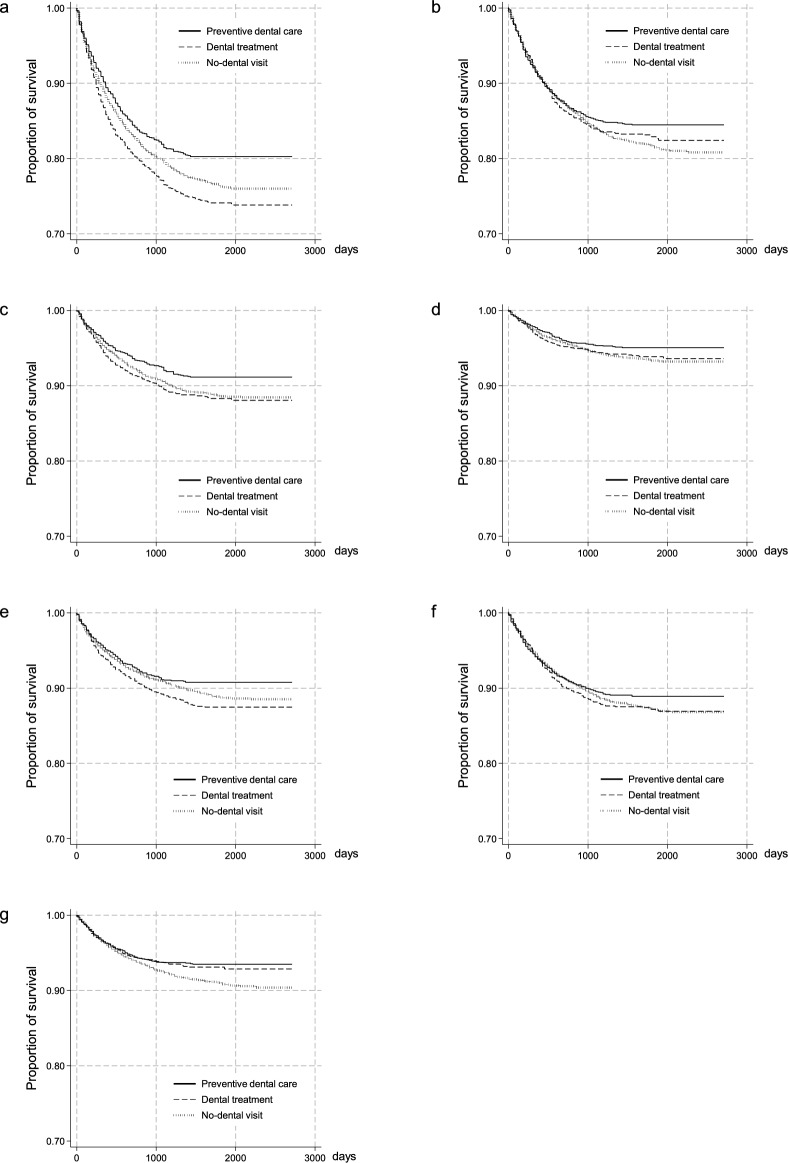
Incidence of CVD (**a**); infectious disease (**b**); acute myocardial infarction (**c**); heart failure (**d**); cerebral infarction (**e**); sepsis (**f**); and pneumonia (**g**) for patients undergoing hemodialysis with no-dental visit, dental treatment, or preventive dental care.

Figure 2a shows the survival curve for the incidence of CVDs stratified by dental care utilization patterns. We found differences in the occurrence of CVD according to dental care utilization patterns (Fig. 2a, p < 0.001 for log-rank test). Although there was no significant difference in the incidence of heart failure (Fig. 2d, p = 0.072 for log-rank test), the incidences of acute myocardial infarction and cerebral infarction differed according to dental care utilization patterns (Fig. 2c,e, p = 0.003 and 0.01 for log-rank test). Tables 2 and 3 show the results of the Cox proportional regression model used to estimate survival time for the incidence of CVD. The preventive dental care group had a lower HR of the incidence of CVD (adjusted hazard ratio [aHR]: 0.86, 95% CI: 0.76–0.97), whereas the dental treatment group had a higher HR of 1.11 (95% CI: 1.00–1.22) compared to the no-dental visit group. The preventive dental care group showed a lower risk for acute myocardial infarction with aHR of 0.79 (95% CI: 0.67–0.93) compared to the no-dental visit group, respectively.

**Table 2:** The effect of dental visit status on primary outcomes; the incidence of CVD and infectious disease.

Outcome	Group	N	Event	Crude risk	Person-Year	Incidence rate	Crude model			Adjusted model*		
							HR	95% CI	p-value	aHR	95% CI	p-value
CVD	All	10,756	2291	0.21	36,978	0.062						
	No-dental visit	6086	1311	0.22	21,299	0.062	Reference			Reference		
	Dental treatment	2196	521	0.24	7008	0.074	1.14	1.03, 1.26	0.014	1.11	1.00, 1.22	0.048
	Preventive dental care	2474	459	0.19	8671	0.053	0.85	0.76, 0.94	0.003	0.86	0.77, 0.96	0.006
Infectious disease	All	10,804	1756	0.16	39,264	0.045						
	No-dental visit	6114	1031	0.17	22,590	0.046	Reference			Reference		
	Dental treatment	2208	356	0.16	7655	0.047	0.97	0.86, 1.10	0.64	0.95	0.84, 1.08	0.45
	Preventive dental care	2482	369	0.15	9019	0.041	0.88	0.78, 0.99	0.038	0.86	0.76, 0.97	0.014

*Adjusted for age, sex, diabetes, hypertension, dementia, chronic pulmonary disease, rheumatic disease, liver disease, hemiplegia, paraplegia, malignancy, metastatic solid tumor, HIV/AIDS, hospitalization in baseline period, and type of the health insurance.

CVD includes acute myocardial infarction, heart failure, and cerebral infarction.

Infectious disease includes sepsis and pneumonia.

**Table 3:** The effect of dental visit status on secondary outcomes; the incidence of acute myocardial infarction, heart failure, cerebral infarction, sepsis, and pneumonia.

Outcome	Group	N	Event	Crude risk	Person-year	Incidence rate	Crude model			Adjusted model*		
							HR	95% CI	p-value	aHR	95% CI	p-value
Acute myocardial infarction	All	10,821	1061	0.10	41,378	0.026						
	No-dental visit	6122	624	0.10	23,806	0.026	Reference			Reference		
	Dental treatment	2209	235	0.11	7974	0.029	1.07	0.92, 1.24	0.39	1.04	0.89, 1.21	0.62
	Preventive dental care	2490	202	0.08	9603	0.021	0.79	0.67, 0.92	0.003	0.79	0.67, 0.93	0.004
Heart failure	All	10,854	608	0.06	42,862	0.014						
	No-dental visit	6145	365	0.06	24,690	0.015	Reference			Reference		
	Dental treatment	2217	126	0.06	8282	0.015	0.98	0.80, 1.19	0.81	0.99	0.68, 1.02	0.76
	Preventive dental care	2492	117	0.05	9890	0.012	0.79	0.64, 0.97	0.023	0.83	0.68, 1.03	0.090
Cerebral infarction	All	10,820	1080	0.10	41,256	0.026						
	No-dental visit	6120	612	0.10	23,812	0.026	Reference			Reference		
	Dental treatment	2208	249	0.11	7884	0.032	1.16	1.00, 1.34	0.053	1.12	0.97, 1.31	0.12
	Preventive dental care	2492	219	0.09	9560	0.023	0.87	0.75, 1.02	0.086	0.88	0.75, 1.03	0.10
Sepsis	All	10,831	1238	0.11	40,890	0.030						
	No-dental visit	6127	712	0.12	23,592	0.030	Reference			Reference		
	Dental treatment	2215	264	0.12	7923	0.033	1.05	0.91, 1.21	0.17	1.02	0.88, 1.17	0.76
	Preventive dental care	2489	262	0.11	9375	0.028	0.91	0.79, 1.04	0.53	0.89	0.78, 1.03	0.13
Pneumonia	All	10,841	801	0.07	42,466	0.019						
	No-dental visit	6138	502	0.08	24,414	0.021	Reference			Reference		
	Dental treatment	2214	143	0.06	8282	0.017	0.80	0.67, 0.97	0.020	0.80	0.66, 0.96	0.019
	Preventive dental care	2489	156	0.06	9769	0.016	0.76	0.64, 0.91	0.003	0.74	0.62, 0.88	0.001

*Adjusted for age, sex, diabetes, hypertension, dementia, chronic pulmonary disease, rheumatic disease, liver disease, hemiplegia, paraplegia, malignancy, metastatic solid tumor, HIV/AIDS, hospitalization in baseline period, and type of the health insurance.

Figure 2b shows the survival curve of the incidence of infectious diseases stratified by dental care utilization patterns. Although there was no significant difference in the incidence of sepsis (Fig. 2f, p = 0.23 for log-rank test), the incidence of pneumonia differed according to dental utilization patterns (Fig. 2g, p = 0.003 for log-rank test). In the preventive dental care group, the aHR for infectious diseases was lower at 0.86 (95% CI: 0.76–0.97) compared to the no-dental visit group (Table 2). The aHR for the incidence of pneumonia in the preventive dental care groups was 0.74 (95% CI: 0.62–0.88) (Table 3). Furthermore, for pneumonia, the dental treatment group showed a lower aHR of 0.80 (95% CI: 0.66–0.96).

The sensitivity analysis conducted by setting different definitions for the outcomes yielded results that were consistent with those of the main analysis (Supplementary Tables 1 and 2). Furthermore, additional analysis, which categorized exposure into four groups including no-dental visit, minor dental treatment, major dental treatment, or preventive dental care groups, yielded results that were consistent with those of the main analysis (Supplementary Tables 3 and 4).

## Discussion

### Summary

This study evaluated the effects of dental care utilization status in patients undergoing hemodialysis. Of the 10,873 patients undergoing hemodialysis extracted from a Japanese claims database, more than half had no-dental visits, 20.4% were in the dental treatment group and approximately 23.0% were in the preventive dental care group. The preventive dental care group had a lower incidence of CVD and infectious diseases. The HR of CVD was higher in the dental treatment group, whereas the HR of pneumonia was lower in the dental treatment group. These findings suggest that appropriate dental care utilization may be beneficial in reducing the risk of CVDs and infectious diseases, which are fatal complications in patients undergoing hemodialysis.

### Comparison with previous studies

In the general population, periodontal disease is significantly associated with CVD^16^. A study using the national health insurance system database in South Korea has shown that severe periodontitis significantly increases the incidence of acute myocardial and cerebral infarctions^17^. However, no clear evidence of the preventive effects of periodontal therapy on the incidence of CVD events exist, although improvements in inflammatory markers and endothelial function have been reported^18^. Meanwhile, in patients undergoing hemodialysis with lower immune function and higher risk of developing CVD events compared with that observed in the general population, periodontal therapy may have a greater impact. In periodontal therapy for patients undergoing hemodialysis, several studies have elucidated the effect of controlling inflammation^19–21^ and preventing cardiovascular events^22^. Two studies using Taiwanese claims data reported that intensive periodontal therapy (scaling and root planing and flap procedure) lowered the risk of infection-related hospitalization^13^, hospitalization due to CVD, and all-cause mortality^3^ in patients undergoing hemodialysis.

However, multidisciplinary dental treatments may be provided in clinical practice because many patients who visit dentists suffer from various dental problems as well as periodontitis. Patients visiting dentists undergo two primary dental treatment modalities. Patients with dental problems should receive the active dental treatments for dental caries, endodontic treatment, tooth extraction, prosthodontic preparation, and intensive periodontal therapy. Patients without major dental problems may receive prophylaxis, including toothbrushing instructions, mechanical tooth polishing, scaling, root planing, and maintenance of the prosthodontic apparatus. This study focused on differences in patients’ dental care utilization patterns. No study has examined how multidisciplinary dental care, not only periodontal therapy, affects the prognosis of patients undergoing hemodialysis. In this study, patients who underwent nonsurgical periodontal therapy were assigned to the preventive dental care group because of its secondary preventive aspect. The results showed a significantly lower HR for the incidence of CVD and infectious diseases in the preventive dental care group were consistent with those of previous studies.

### Possible explanations

Some possible mechanisms by which professional dental care affects CVD and infectious diseases in patients undergoing hemodialysis can be assumed, although detailed information on the participants, such as blood and bacteriological test results, were not available in this study. In general, the mechanisms by which oral health affects systemic health include the impact of local inflammation in the oral cavity due to dental diseases, systemic repercussions of the local inflammation through circulation, and influence of dental plaque containing abundant oral bacteria. In addition to these, specific mechanisms pertinent to patients undergoing dialysis may include the mediating effect of malnutrition–inflammation–atherosclerosis (MIA) syndrome.

Patients with periodontitis have systemic inflammation with raised levels of high-sensitivity C-reactive protein (CRP)^23–25^, and periodontal treatments reduce this inflammatory reaction^19–21^. A high CRP level is a decisive risk factor for CVD events^26^, and previous cohort studies have reported that the presence of periodontitis increases mortality from CVD in patients undergoing hemodialysis^6,7^. Therefore, well-achieved periodontal therapy has the potential to contribute to reducing the incidence of CVD. Regarding heart failure and cerebral infarction, a tendency toward reduced incidence risk was observed with preventive dental care, although no significant effects were found. CVDs are influenced by various factors including oral health, as well as lifestyle habits and environmental factors. Hence, this study’s sample size was likely insufficient to detect an impact of oral health.

In patients undergoing hemodialysis, the risks of infections including nosocomial infections are heightened. These risks are not only because these patients are immunocompromised but also because their rate of exposure to infection risks, such as hospitalizations, frequent outpatient visits^27^, and the placement of vascular access devices^28^, is high. Reducing the total number of bacteria in the oral cavity can help prevent infectious diseases. Poor oral health may increase the number of bacteria transferred into the bloodstream through daily oral activities such as tooth brushing and chewing^29^. These bacteria can trigger bacteremia and, eventually, sepsis in patients undergoing hemodialysis. However, in this study, dental care did not significantly reduce the risk of sepsis. Further larger-scale studies might detect the statistically significant effect of oral care on sepsis. Reducing the number of oral bacteria can prevent aspiration pneumonia^30–32^. Considering that patients undergoing hemodialysis are an immunocompromised population, preventive dental care may lower the incidence of pneumonia. Interestingly, a significantly lower incidence of pneumonia was observed not only in the preventive dental care group but also in the dental treatment group. Because we could not conduct stratified analyses for each type of dental treatment due to limited sample size, which specific dental treatments are associated with this lower incidence remains unclear. However, a reduction in oral bacterial load following caries treatment or extractions, professional denture cleaning^33,34^, or improved nutritional status through enhanced chewing efficiency following prosthodontic treatment^35^ could have enhanced immune responses. The additional analysis in this study suggests that major dental treatments involving pulpectomies, periodontal surgeries, or tooth extractions can be effective in reducing the risk of pneumonia.

Poor oral health may affect the incidence of CVD and infectious diseases through the MIA syndrome^36,37^. Some patients undergoing hemodialysis experience MIA syndrome, which is a combination of chronic systemic inflammation, malnutrition, and arteriosclerosis^38^. Oral problems, such as pain, ill-fitting dentures, and missing teeth with no prosthodontic treatment, may enhance malnutrition in patients undergoing hemodialysis patients^39–41^. Periodontitis increases systemic inflammation^23–25^. Malnutrition and inflammation proceed in a mutually conducive vicious cycle, leading to the risk of atherosclerosis and infections due to a depressed immune system in patients undergoing hemodialysis^42^.

Differences in the effects of dental treatments on the incidence of CVD and infectious diseases have been reported. The dental treatment visit group included people who visited for dental problems rather than for maintenance. The effect of poor oral health on the cardiovascular system before the baseline period may have been reflected in the significantly higher HR for CVD in the dental treatment group. The dental treatment group may represent a subpopulation with different health-related behaviors compared to the other groups, and this divergence in health-related behavior could potentially contribute to the observed results. In contrast, for pneumonia, when the number of oral bacteria is reduced by dental treatment, the effect may have been immediately apparent in the reduction in the risk of aspiration pneumonia.

### Implications

In this study, a significant association was observed between preventive dental care and the incidence of CVD and infectious diseases. These findings suggest that preventive dental care, including prophylactic programs and maintenance of the prosthodontic apparatus, may be beneficial in patients undergoing hemodialysis. Oral care can reduce the risk of aspiration pneumonia in the older residents of nursing homes^32^, and that of cardiovascular diseases in the Scottish general population with a mean age of 50.0 years^43^. Patients undergoing hemodialysis are more susceptible to cardiovascular disease and infectious diseases, and have poor oral health. Therefore, the impact of dental utilization in our study population may have been more pronounced than in the general population. Effective prevention of CVD includes control of comorbidities, such as hypertension and conditions that cause inflammation, while prevention of infectious diseases includes primary infection control and vaccination. In addition, dental care may serve as a preventive measure. Patients undergoing hemodialysis have poor oral health^44–46^ because of salivary gland atrophy and drinking restrictions^45^. However, they were unlikely to visit dental clinics because of anxiety about dental treatment and financial concerns^47,48^. This study suggests that dental care provided by dental health professionals is beneficial for preventing fatal complications. Healthcare professionals should establish a system in which patients undergoing hemodialysis can receive preventive dental care without concern.

Dental visits, either dental treatment or preventive dental care, are associated with a lower risk of pneumonia, possibly owing to a reduction in oral bacteria. However, the finding that dental treatment is associated with a higher OR of the incidence of CVD needs to be examined in more detail in the future, including the type of treatment and the duration of time to CVD events.

### Strength and limitations

To the best of our knowledge, this is the first study to demonstrate that regular dental visits may be beneficial in the primary prevention of CVDs and infectious diseases in patients with ESRD undergoing hemodialysis. Another strength of this study is that our longitudinal claims data included a large sample size without selection bias. Utilizing real-world data, we generated real-life clinical evidence for patients with ESRD undergoing hemodialysis.

However, this study also had several limitations. First, several potentially confounding factors, such as body mass index, smoking status, severity of periodontitis, unadjusted comorbidities that may affect physical activity, and socioeconomic status, were unavailable in the database. Regarding socioeconomic status, we adjusted for the type of health insurance as a proxy for income and educational background due to its correlation with income^49^. The data regarding income nor educational background were not available in the database used in this study. Second, our findings require careful interpretation for application to populations of different races or other dialysis modalities, such as peritoneal dialysis. Third, individuals without an observation period of 1 year after the initiation of hemodialysis were excluded in this study, potentially introducing survivor bias. This means artificially selecting a population with better health status, which may have led to an underestimation of the actual hazard ratios. Additionally, the no-dental visit group may include individuals who refrained from dental visits despite their poor oral health and those who refrained due to their good oral health. While this might be a potential bias, it may introduce variability in the results rather than skew them in a particular direction, thus ensuring the robustness of the study findings. Fourth, individuals were categorized into three groups based on their dental visit history during the baseline period, and each group was considered to exhibit a different dental utilization behavior. However, dental utilization behavior may change during the lifetime, and the potential effects of such changes require further investigation in future research. Furthermore, we did not account for dental visit behaviors prior to the baseline period. While the preventive dental care group may include individuals who received preventive dental care after undergoing significant dental procedures such as tooth extraction before the baseline period, we believe they are equivalent to those who continued preventive dental care without significant dental procedures prior to the baseline period, provided their oral health had improved by the start of the baseline period. Thus, to enhance the interpretability of the study findings, allocations were based solely on dental visit behaviors during the baseline period. Finally, the disease and procedure codes in the claims data were consistent. Previous studies that examined the validity of Japanese claims data demonstrated their usefulness in research using administrative data^50–52^. In Japan, no procedure code links directly to disease codes, leading to both dental treatments and preventive dental care being provided under the same disease codes. Therefore, exposure was defined based on dental procedure codes according to a previous study^53^.

## Conclusion

This study demonstrated that dental visits for preventive dental care were associated with a significant risk reduction of CVD and infectious complications in patients with ESRD undergoing hemodialysis. Healthcare professionals should be aware of oral health issues and promote the patients' dental visits.

### Supplementary Information

Supplementary Figure 1.

Supplementary Legends.

Supplementary Tables.

## Data Availability

The data that support the findings of this study are available from DeSC Healthcare Inc. but restrictions apply to the availability of these data, which were used under license for the current study, and so are not publicly available. Data are however available from the authors upon reasonable request and with permission of DeSC Healthcare Inc.
