# Supplementary figures and images for: Preventive dental care reduces risk of cardiovascular disease and pneumonia in hemodialysis population: a nationwide claims database analysis
Source: Sci Rep. 2024 May 29;14:12372. doi: 10.1038/s41598-024-62735-3 (PMC11137030; doi:10.1038/s41598-024-62735-3)

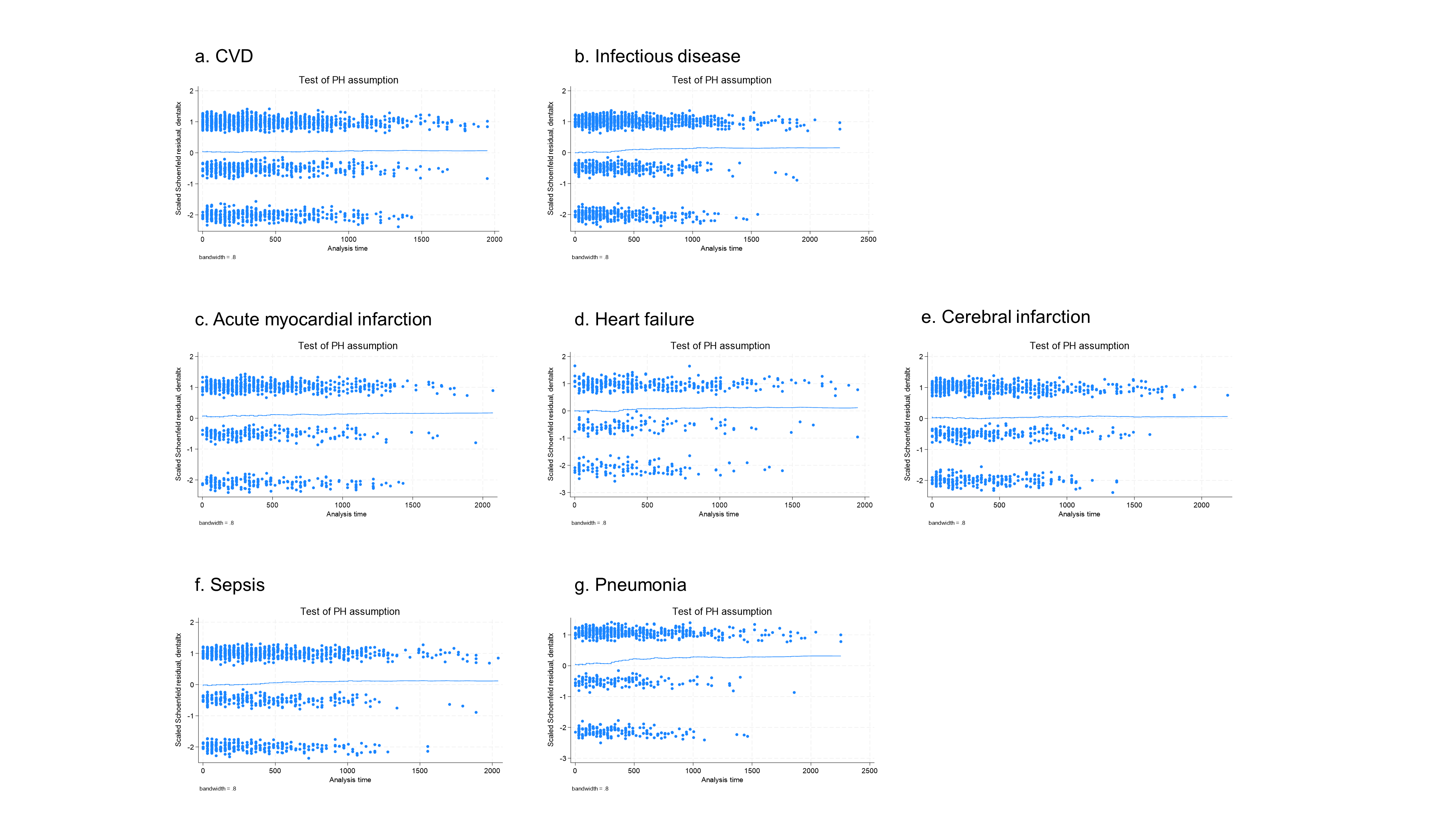

Supplement: Supplementary file 1 — Supplementary Figure 1. [file 41598_2024_62735_MOESM1_ESM.tif]
