# Supplementary material for: Preventive dental care reduces risk of cardiovascular disease and pneumonia in hemodialysis population: a nationwide claims database analysis
Source: Sci Rep. 2024 May 29;14:12372. doi: 10.1038/s41598-024-62735-3 (PMC11137030; doi:10.1038/s41598-024-62735-3)
Supplement: Supplementary file 2 — Supplementary Legends. [file 41598_2024_62735_MOESM2_ESM.docx]

**Supplementary figure legend**

Supplementary Figure 1

The proportional hazards assumption test of the Cox proportional regression models. The proportional hazards assumption was confirmed using graphical diagnostics based on Schoenfeld residuals plots.
