# Supplementary material for: Preventive dental care reduces risk of cardiovascular disease and pneumonia in hemodialysis population: a nationwide claims database analysis
Source: Sci Rep. 2024 May 29;14:12372. doi: 10.1038/s41598-024-62735-3 (PMC11137030; doi:10.1038/s41598-024-62735-3)
Supplement: Supplementary file 3 — Supplementary Tables. [file 41598_2024_62735_MOESM3_ESM.docx]

| Supplementary Table 1. Sensitivity analysis on the effect of dental visit status on primary outcomes; the incidence of CVD and infectious disease | | | | | | | | | | | | | | |
| --- | --- | --- | --- | --- | --- | --- | --- | --- | --- | --- | --- | --- | --- | --- |
| **Outcome** | **Group** | **N** | **Event** | **Crude risk** | **Person-Year** | **Incidence rate** |  | **Crude model** | | |  | **Adjusted model*** | | |
|  |  |  |  |  |  |  |  | **HR** | **95%CI** | **p-value** |  | **aHR** | **95%CI** | **p-value** |
| CVD | All | 10756 | 2133 | 0.20 | 36978 | 0.058 |  |  |  |  |  |  |  |  |
|  | No-dental visit | 6086 | 1226 | 0.20 | 8671 | 0.141 |  | Reference | | |  | Reference | | |
|  | Dental treatment | 2196 | 488 | 0.22 | 7011 | 0.070 |  | 1.14 | 1.02, 1.26 | 0.016 |  | 1.11 | 0.997, 1.23 | 0.056 |
|  | Preventive dental care | 2474 | 419 | 0.17 | 21299 | 0.020 |  | 0.83 | 0.74, 0.93 | 0.001 |  | 0.84 | 0.75, 0.94 | 0.002 |
|  |  |  |  |  |  |  |  |  |  |  |  |  |  |  |
| Infectious disease | All | 10804 | 1191 | 0.11 | 39264 | 0.030 |  |  |  |  |  |  |  |  |
|  | No-dental visit | 6114 | 710 | 0.12 | 22590 | 0.031 |  | Reference | | |  | Reference | | |
|  | Dental treatment | 2208 | 228 | 0.10 | 7655 | 0.030 |  | 0.90 | 0.77, 1.05 | 0.178 |  | 0.89 | 0.77, 1.03 | 0.127 |
|  | Preventive dental care | 2482 | 253 | 0.10 | 9019 | 0.028 |  | 0.88 | 0.76, 1.01 | 0.076 |  | 0.85 | 0.74. 0.98 | 0.027 |
| *: Adjusted for age, sex, diabetes, hypertension, dementia, chronic pulmonary disease, rheumatic disease, liver disease, hemiplegia, paraplegia, malignancy, metastatic solid tumor, HIV/AIDS, hospitalization in baseline period, and type of the health insurance | | | | | | | | | | | | | | |
| CVD includes acute myocardial infarction, heart failure, and cerebral infarction. | | | | | | | | | | | | | | |
| Infectious disease includes sepsis and pneumonia. | | | | | | | | | | | | | | |

| Supplementary Table 2. Sensitivity analysis on the effect of dental visit status on secondary outcomes; the incidence of acute myocardial infarction, heart failure, cerebral infarction, sepsis, and pneumonia | | | | | | | | | | | | | | |
| --- | --- | --- | --- | --- | --- | --- | --- | --- | --- | --- | --- | --- | --- | --- |
| **Outcome** | **Group** | **N** | **Event** | **Crude risk** | **Person-Year** | **Incidence rate** |  | **Crude model** | | |  | **Adjusted model*** | | |
|  |  |  |  |  |  |  |  | **HR** | **95%CI** | **p-value** |  | **aHR** | **95%CI** | **p-value** |
| Acute myocardial infarction | All | 10821 | 439 | 0.04 | 41378 | 0.011 |  |  |  |  |  |  |  |  |
|  | No-dental visit | 6122 | 272 | 0.04 | 23800 | 0.011 |  | Reference | | |  | Reference | | |
|  | Dental treatment | 2209 | 92 | 0.04 | 7974 | 0.012 |  | 0.96 | 0.75, 1.21 | 0.71 |  | 0.93 | 0.73, 1.18 | 0.54 |
|  | Preventive dental care | 2490 | 75 | 0.03 | 9603 | 0.008 |  | 0.67 | 0.52, 0.87 | 0.002 |  | 0.68 | 0.52, 0.88 | 0.003 |
|  |  |  |  |  |  |  |  |  |  |  |  |  |  |  |
| Heart failure | All | 10854 | 462 | 0.04 | 42862 | 0.011 |  |  |  |  |  |  |  |  |
|  | No-dental visit | 6145 | 277 | 0.05 | 24690 | 0.011 |  | Reference | | |  | Reference | | |
|  | Dental treatment | 2217 | 96 | 0.04 | 8282 | 0.012 |  | 0.98 | 0.77. 1.23 | 0.85 |  | 0.97 | 0.77, 1.23 | 0.81 |
|  | Preventive dental care | 2492 | 89 | 0.04 | 9890 | 0.009 |  | 0.79 | 0.62, 1.00 | 0.051 |  | 0.84 | 0.66, 1.07 | 0.16 |
|  |  |  |  |  |  |  |  |  |  |  |  |  |  |  |
| Cerebral infarction | All | 10820 | 627 | 0.06 | 41256 | 0.015 |  |  |  |  |  |  |  |  |
|  | No-dental visit | 6120 | 351 | 0.06 | 23812 | 0.015 |  | Reference | | |  | Reference | | |
|  | Dental treatment | 2208 | 147 | 0.07 | 7884 | 0.019 |  | 1.19 | 0.98, 1.44 | 0.083 |  | 1.16 | 0.95, 1.40 | 0.14 |
|  | Preventive dental care | 2492 | 129 | 0.05 | 9560 | 0.013 |  | 0.90 | 0.73, 1.10 | 0.29 |  | 0.90 | 0.73, 1.11 | 0.32 |
|  |  |  |  |  |  |  |  |  |  |  |  |  |  |  |
| Sepsis | All | 10831 | 679 | 0.06 | 40890 | 0.017 |  |  |  |  |  |  |  |  |
|  | No-dental visit | 6127 | 398 | 0.06 | 23592 | 0.017 |  | Reference | | |  | Reference | | |
|  | Dental treatment | 2215 | 142 | 0.06 | 7923 | 0.018 |  | 1.01 | 0.83, 1.22 | 0.951 |  | 1.16 | 0.95, 1.40 | 0.32 |
|  | Preventive dental care | 2489 | 139 | 0.06 | 9375 | 0.015 |  | 0.86 | 0.71, 1.04 | 0.128 |  | 0.90 | 0.73, 1.11 | 0.14 |
|  |  |  |  |  |  |  |  |  |  |  |  |  |  |  |
| Pneumonia | All | 10841 | 570 | 0.05 | 42466 | 0.013 |  |  |  |  |  |  |  |  |
|  | No-dental visit | 6138 | 354 | 0.06 | 24414 | 0.014 |  | Reference | | |  | Reference | | |
|  | Dental treatment | 2214 | 98 | 0.04 | 8282 | 0.012 |  | 0.78 | 0.62, 0.97 | 0.028 |  | 0.78 | 0.62, 0.97 | 0.027 |
|  | Preventive dental care | 2489 | 118 | 0.05 | 9769 | 0.012 |  | 0.82 | 0.67, 1.00 | 0.062 |  | 0.79 | 0.64, 0.97 | 0.024 |
| *: Adjusted for age, sex, diabetes, hypertension, dementia, chronic pulmonary disease, rheumatic disease, liver disease, hemiplegia, paraplegia, malignancy, metastatic solid tumor, HIV/AIDS, hospitalization in baseline period, and type of the health insurance | | | | | | | | | | | | | | |

| Supplementary Table 3. The effect of dental visit status, accounting for dental procedure, on primary outcomes; the incidence of CVD and infectious disease | | | | | | | | | | | | | | |
| --- | --- | --- | --- | --- | --- | --- | --- | --- | --- | --- | --- | --- | --- | --- |
| **Outcome** | **Group** | **N** | **Event** | **Crude risk** | **Person-Year** | **Incidence rate** |  | **Crude model** | | |  | **Adjusted model*** | | |
|  |  |  |  |  |  |  |  | **HR** | **95%CI** | **p-value** |  | **aHR** | **95%CI** | **p-value** |
| CVD | All | 10756 | 2291 | 0.21 | 36978 | 0.062 |  |  |  |  |  |  |  |  |
|  | No-dental visit | 6086 | 1311 | 0.22 | 21299 | 0.062 |  | Reference | | |  | Reference | | |
|  | Minor dental treatment | 1257 | 302 | 0.24 | 3912 | 0.077 |  | 1.16 | 1.02, 1.31 | 0.020 |  | 1.13 | 1.00, 1.29 | 0.049 |
|  | Major dental treatment | 939 | 219 | 0.23 | 3096 | 0.071 |  | 1.11 | 0.96, 1.28 | 0.17 |  | 1.07 | 0.93, 1.24 | 0.33 |
|  | Preventive dental care | 2474 | 459 | 0.19 | 8671 | 0.053 |  | 0.85 | 0.76, 0.94 | 0.003 |  | 0.86 | 0.77, 0.96 | 0.006 |
|  |  |  |  |  |  |  |  |  |  |  |  |  |  |  |
| Infectious disease | All | 10804 | 1756 | 0.16 | 39264 | 0.045 |  |  |  |  |  |  |  |  |
|  | No-dental visit | 6114 | 1031 | 0.17 | 22590 | 0.046 |  | Reference | | |  | Reference | | |
|  | Minor dental treatment | 1271 | 196 | 0.15 | 4330 | 0.045 |  | 0.93 | 0.80, 1.09 | 0.38 |  | 0.92 | 0.78, 0.96 | 0.26 |
|  | Major dental treatment | 937 | 160 | 0.17 | 3325 | 0.048 |  | 1.02 | 0.87, 1.21 | 0.8 |  | 1.01 | 0.85, 1.19 | 0.95 |
|  | Preventive dental care | 2482 | 369 | 0.15 | 9019 | 0.041 |  | 0.88 | 0.78, 0.99 | 0.038 |  | 0.86 | 0.76, 0.97 | 0.014 |
| *: Adjusted for age, sex, diabetes, hypertension, dementia, chronic pulmonary disease, rheumatic disease, liver disease, hemiplegia, paraplegia, malignancy, metastatic solid tumor, HIV/AIDS, hospitalization in baseline period, and type of the health insurance | | | | | | | | | | | | | | |
| CVD includes acute myocardial infarction, heart failure, and cerebral infarction. | | | | | | | | | | | | | | |
| Infectious disease includes sepsis and pneumonia. | | | | | | | | | | | | | | |

| Supplementary Table 4. The effect of dental visit status, accounting for dental procedure, on secondary outcomes; the incidence of acute myocardial infarction, heart failure, cerebral infarction, sepsis, and pneumonia | | | | | | | | | | | | | | |
| --- | --- | --- | --- | --- | --- | --- | --- | --- | --- | --- | --- | --- | --- | --- |
| **Outcome** | **Group** | **N** | **Event** | **Crude risk** | **Person-Year** | **Incidence rate** |  | **Crude model** | | |  | **Adjusted model*** | | |
|  |  |  |  |  |  |  |  | **HR** | **95%CI** | **p-value** |  | **aHR** | **95%CI** | **p-value** |
| Acute myocardial infarction | All | 10821 | 1061 | 0.10 | 41378 | 0.026 |  |  |  |  |  |  |  |  |
|  | No-dental visit | 6122 | 624 | 0.10 | 23800 | 0.026 |  | Reference | | |  | Reference | | |
|  | Minor dental treatment | 1268 | 138 | 0.11 | 4455 | 0.031 |  | 1.10 | 0.92, 1.32 | 0.30 |  | 1.08 | 0.89, 1.29 | 0.44 |
|  | Major dental treatment | 941 | 97 | 0.10 | 3519 | 0.028 |  | 1.02 | 0.83, 1.27 | 0.84 |  | 0.99 | 0.80, 1.23 | 0.93 |
|  | Preventive dental care | 2490 | 202 | 0.08 | 9603 | 0.021 |  | 0.79 | 0.67, 0.92 | 0.003 |  | 0.79 | 0.68, 0.93 | 0.004 |
|  |  |  |  |  |  |  |  |  |  |  |  |  |  |  |
| Heart failure | All | 10854 | 608 | 0.06 | 42862 | 0.014 |  |  |  |  |  |  |  |  |
|  | No-dental visit | 6145 | 365 | 0.06 | 24690 | 0.015 |  | Reference | | |  | Reference | | |
|  | Minor dental treatment | 1274 | 75 | 0.06 | 4631 | 0.016 |  | 1.02 | 0.80, 1.31 | 0.88 |  | 1.01 | 0.79, 1.29 | 0.95 |
|  | Major dental treatment | 943 | 51 | 0.05 | 3652 | 0.014 |  | 0.92 | 0.68, 1.23 | 0.56 |  | 0.91 | 0.68, 1.23 | 0.55 |
|  | Preventive dental care | 2492 | 117 | 0.05 | 9890 | 0.012 |  | 0.79 | 0.64, 0.97 | 0.023 |  | 0.83 | 0.68, 1.02 | 0.090 |
|  |  |  |  |  |  |  |  |  |  |  |  |  |  |  |
| Cerebral infarction | All | 10820 | 1080 | 0.10 | 41256 | 0.026 |  |  |  |  |  |  |  |  |
|  | No-dental visit | 6120 | 612 | 0.10 | 23812 | 0.026 |  | Reference | | |  | Reference | | |
|  | Minor dental treatment | 1266 | 148 | 0.12 | 4428 | 0.033 |  | 1.21 | 1.01, 1.44 | 0.041 |  | 1.17 | 0.98, 1.40 | 0.083 |
|  | Major dental treatment | 942 | 101 | 0.11 | 3456 | 0.029 |  | 1.09 | 0.88, 1.34 | 0.42 |  | 1.06 | 0.86, 1.31 | 0.58 |
|  | Preventive dental care | 2492 | 219 | 0.09 | 9560 | 0.023 |  | 0.87 | 0.75, 1.02 | 0.086 |  | 0.88 | 0.75, 1.03 | 0.10 |
|  |  |  |  |  |  |  |  |  |  |  |  |  |  |  |
| Sepsis | All | 10831 | 1238 | 0.11 | 40890 | 0.030 |  |  |  |  |  |  |  |  |
|  | No-dental visit | 6127 | 712 | 0.12 | 23592 | 0.030 |  | Reference | | |  | Reference | | |
|  | Minor dental treatment | 1276 | 134 | 0.11 | 4512 | 0.030 |  | 0.92 | 0.77, 1.11 | 0.39 |  | 0.9 | 0.75, 1.09 | 0.28 |
|  | Major dental treatment | 939 | 130 | 0.14 | 3411 | 0.038 |  | 1.22 | 1.01, 1.47 | 0.041 |  | 1.18 | 0.98, 1.43 | 0.077 |
|  | Preventive dental care | 2489 | 262 | 0.11 | 9375 | 0.028 |  | 0.90 | 0.79, 1.04 | 0.17 |  | 0.90 | 0.78, 1.03 | 0.13 |
|  |  |  |  |  |  |  |  |  |  |  |  |  |  |  |
| Pneumonia | All | 10841 | 801 | 0.07 | 42466 | 0.019 |  |  |  |  |  |  |  |  |
|  | No-dental visit | 6138 | 502 | 0.08 | 24414 | 0.021 |  | Reference | | |  | Reference | | |
|  | Minor dental treatment | 1272 | 85 | 0.07 | 4637 | 0.018 |  | 0.84 | 0.67, 1.05 | 0.13 |  | 0.83 | 0.66, 1.05 | 0.114 |
|  | Major dental treatment | 942 | 58 | 0.06 | 3645 | 0.016 |  | 0.75 | 0.57, 0.99 | 0.042 |  | 0.76 | 0.58, 0.998 | 0.048 |
|  | Preventive dental care | 2489 | 156 | 0.06 | 9769 | 0.016 |  | 0.76 | 0.64, 0.91 | 0.003 |  | 0.74 | 0.62, 0.88 | 0.001 |
| *: Adjusted for age, sex, diabetes, hypertension, dementia, chronic pulmonary disease, rheumatic disease, liver disease, hemiplegia, paraplegia, malignancy, metastatic solid tumor, HIV/AIDS, hospitalization in baseline period, and type of the health insurance | | | | | | | | | | | | | | |
